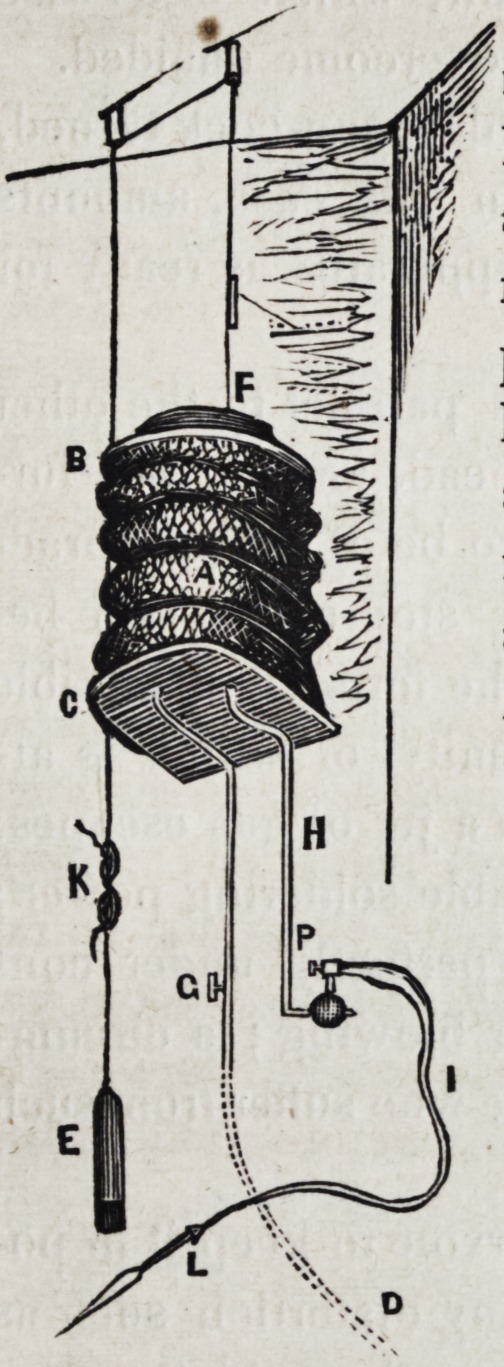# On a New Soldering Apparatus

**Published:** 1852-07

**Authors:** Spence Bate


### ARTICLE IX.

*On a New Soldering Apparatus.* By Spence Bate.

A short time since being on a visit at my father's residence, who practices at Plymouth, I was struck with the utility and effective adaptation of an apparatus for soldering, which is calculated to supersede the blow-pipe and lamp, as being less expensive in work, more powerful and easy of performing its duty, while it relieves the workman from the wearying, and, perchance, hurtful task of using his lungs as a pair of bellows. It is upon these accounts I believe it to be worthy of being communicated to the profession, as a valuable adjunct to the mechanical department, and therefore avail myself of the pages of the Journal of Dental Science, for that purpose.

The apparatus consists of a large sac or resevoir (A) made of vulcanized India rubber, covered externally with a net, the top and bottom (B and C) side figure, being a flat piece of wood, cut round upon the outer side, but square where it is brought in contact with the wall against which it is fixed. The lower of these is stationary, while the upper is movable, thereby constituting a bellows, which, instead of being filled with air, is charged with coal-gas.

Through the bottom or floor of the reservoir are made two holes, to which are connected pipes—the one being in continuation with those which supply the rest of the house, is an inlet; the other being connected with a flexible tube and blowpipe, is an outlet passage. The inlet pipe has a stop-cock (P) which is used in order to secure the gas against returning, and passes through an ivory channel, where the reservoir is full, in order to facilitate the injection of which, an iron rod, connected with a rope pulley, is attached to a circular board which forms the top or roof of the reservoir; at the opposite extremity is appended to the rope, a weight (E) which assists in overcoming the atmospheric pressure upon the reservoir, which otherwise is too great for the pressure of the gas to overcome unaided.

The reservoir being charged, and the inlet stop-cock turned, it is necessary to place a weight, which, in this case, amounts to about 28 lbs. on the top, and the apparatus is ready for use.

From the reservoir, the outlet pipe runs parallel to the other for a short distance, where it receives a head, and is also furnished with a movable joint to enable it to be of greater practical value; attached to which is another stop-cock, (P,) beyond this point the pipe is continued in the form of a flexible tube, made of India rubber, to the extremity of which is attached a simple blow-pipe, through which a jet of gas escapes, which, upon being lighted, makes a valuable soldering power; the quantity and force of the flame being perfectly under control, without the assistance of any lamp or blowing; a circumstance which must be appreciated by those who suffer from such kind of exertion.

There is a wooden ring within the reservoir to keep it in position, and the net outside also prevents any distortion such as might result from the inequality of the strength or thickness of the India rubber.

When the apparatus is required for use, the weight (E) should be belayed to the point (K,) or better still removed, when not in use it should be left suspended, or the pressure of the weights at (F) would drive the gas back, though the stop-cock (I) may be turned.

When the reservoir is empty and require to be filled, it is necessary to remove the weight on the top (F) or, perhaps, which may do equally as well, an equal number of pounds may be added, additional, to the suspended weight at (E;) otherwise the gas has not sufficient power to inject the sac.

The cost of the whole apparatus is about £3. £2.10 for materials, and 10*s*. for labor in fixing it up.

The economy appears to be much over that of common oil to use.

The reservoir contains two square feet of gas, which, at the price of four shillings per thousand feet, may be filled 125 times for two shillings, each time, being capable of doing all the blow-pipe and soldering work for a simple set, and is capable of melting brass wire the one-eighth of an inch in diameter.

Since my attention has been drawn to the apparatus here described, I have heard of a blow-pipe used in a chemical work, some miles from this town, wherein hydrogen is the gas employed, and so perfect a deoxydiser is hydrogen, that two neutrals, gold and silver, for instance, will unite without the assistance of flux or solder, and so powerful is the heat, that it will solder substances, far out of the reach of ordinary blow-pipes, and since hydrogen is capable of being obtained at a price nominally insignificant, I am inclined to think it will be an agent most capable of superseding others now in use. I am about to have one fitted up in my work-room, the result of which, after a fair trial, I shall feel happy to communicate to the pages of the Journal of Dental Science.

A, The Reservoir.

B, Top made of circular piece of wood.

C, Bottom made of wood fixed to the wall.

D, Inlet pipe connected with public reservoir.

G, Stop-cock to preclude the return of gas.

H, Outlet pipe.

I, Flexible tube, made of vulcanised India rubber.

L, Blow-pipe.

F, Weight, to force the gas through the outlet pipe when required.

E, Weight suspended to counteract, when required, the pressure at F.

## Figures and Tables

**Figure f1:**